# The increased cfRNA of TNFSF4 in peripheral blood at late gestation and preterm labor: its implication as a noninvasive biomarker for premature delivery

**DOI:** 10.3389/fimmu.2023.1154025

**Published:** 2023-05-18

**Authors:** Zhe Wang, Qingjian Ou, Lu Gao

**Affiliations:** ^1^ Department of Physiology, College of Basic Medical Sciences, Naval Medical University, Shanghai, China; ^2^ Department of Ophthalmology of Tongji Hospital, School of Medicine, Tongji University, Shanghai, China; ^3^ Shanghai Key Laboratory for Assisted Reproduction and Reproductive Genetics, Shanghai Jiaotong University, Shanghai, China

**Keywords:** cell-free RNA, gestational age (GA), preterm labor, noninvasive biomarkers, inflammation, maternal-fetal interface

## Abstract

**Introduction:**

Given the important roles of immune tolerance and inflammation in both preterm and term labor, some inflammation-related genes could be related to the initiation of labor, even preterm labor. Inspection of cell-free RNA (cfRNA) engaged in inflammation in maternal blood may represent the varied gestational age and may have significant implications for the development of noninvasive diagnostics for preterm birth.

**Methods:**

To identify potential biomarkers of preterm birth, we investigated the cfRNA and exosomal miRNA in the peripheral blood of pregnant women at different gestational ages that undergo term labor or preterm labor. 17 inflammatory initiation-related cfRNAs were screened by overlapping with the targets of decreasing miRNAs during gestation and highly expressed cfRNAs at late gestation in maternal blood. To reveal the origins and mechanisms of these screened cfRNAs, the datasets of single-cell RNA sequencing from peripheral blood mononuclear cells of pregnant women, the fetal lung, and the placenta across different gestational ages were analyzed.

**Results:**

During late gestation, TNFSF4 expression increased exclusively in pro-inflammatory macrophages of maternal blood, whereas its receptor, TNFRSF4, increased expression in T cells from the decidua, which suggested the potential cell-cell communication of maternally-originated pro-inflammatory macrophages with the decidual T cells and contributed to the initiation of labor. Additionally, the cfRNA of TNFSF4 was also increased in preterm labor compared to term labor in the validation cohorts. The EIF2AK2 and TLR4 transcripts were increased in pro-inflammatory macrophages from both fetal lung and placenta but not in those from maternal mononuclear cells at late gestation, suggesting these cfRNAs are possibly derived from fetal tissues exclusively. Moreover, EIF2AK2 and TLR4 transcripts were found highly expressed in the pro-inflammatory macrophages from decidua as well, which suggested these specific fetal-origin macrophages may function at the maternal-fetal interface to stimulate uterine contractions, which have been implicated as the trigger of parturition and preterm labor.

**Discussion:**

Taken together, our findings not only revealed the potential of peripheral TNFSF4 as a novel cfRNA biomarker for noninvasive testing of preterm labor but further illustrated how maternal and fetal signals coordinately modulate the inflammatory process at the maternal-fetal interface, causing the initiation of term or preterm labor.

## 1 Introduction

The non-invasive detection of cell-free RNA (cfRNA) transcripts from pregnant peripheral blood, which may originate from either fetal tissues or maternal tissues, is ideal blood tests to provide diagnostic indications for fetal genetic risk, gestational age and estimate the risk of preterm labor (1–5). Screening specific and efficient cfRNA markers can increased the accuracy and wide application of non-invasive genetic testing for pregnancy. Preterm birth (<37 completed weeks of gestation), the leading cause of neonatal death and the second most common cause of death in children under 5 years old, is responsible for 0.66 million neonates to die (6). The common assumption of parturition is that preterm labor is similar to term labor; the main difference between them is when labor begins. Common to both preterm and term labor is the activation of the myometrium, which is accompanied by the transition from anti-inflammation to pro-inflammation, linking the unique pathways of chemokines, cytokines, and inflammasomes (7). Thus, the exploration of specific cfRNA markers could help to predict and early intervene preterm labor.

Predicting preterm labor could enhance prenatal care, lower the incidence of premature birth, and improve the prognosis for newborns. Ultrasound imaging and the estimation of the last menstrual cycle are typical approaches for estimating gestational age; however, they do not account for preterm labor. Arisoy et al. suggested the measurement of cervicovaginal fetal fibronectin (fFN) and transvaginal sonographic cervical length (CL) have low positive predictive values (17% for fFN and 21% for CL) in clinical studies (8). Recently, the exploration of novel biomarkers for noninvasive detection has gained more and more attention, which provides new clues for the prediction and early diagnosis of preterm labor (1, 2). MicroRNAs (miRNAs) from maternal or fetal tissues (such as placenta) can be released into the peripheral blood, which has higher stability, sensitivity, and specificity than traditional protein biomarkers. Since the miRNAs from exosomes can enter into target cells and tissues to regulate the gene expression of and indicate the status of pregnancy and the pregnancy complications (9–12), they have been considered as new fingerprint of the pregnant progression and state (9, 13, 14).

Previous studies suggested the inflammatory initiation and activation of the immune microenvironment at the maternal-fetal interface (including placental trophoblast cells, decidual cells, and immune cells) can facilitate the initiation of term or preterm labor (7, 15, 16). For instance, the transition from anti-inflammatory to pro-inflammatory signaling in activated macrophages can augment uterine contraction, resulting in preterm parturition (3, 16). The spreading and homing of inflammatory neutrophils and macrophages in the maternal-fetal interface is facilitated by chemokines and cellular adhesion molecules. Additionally, the NLRP3 inflammasome is essential for the onset of labor. NLRP3 also regulates the functional status of neutrophils and macrophages in the uterus and decidua, without altering their influx, as well as maternal systemic inflammation (17–19). Finally, the increased number of activated T cells may enhance the production of cytokines and interferon, and promote the inflammatory process by recruiting macrophages to the decidua, which causes the degradation of the collagen in the fetal membrane and the extracellular matrix in the cervix, and ultimately results in preterm labor (16, 20, 21).

**Figure 1:**
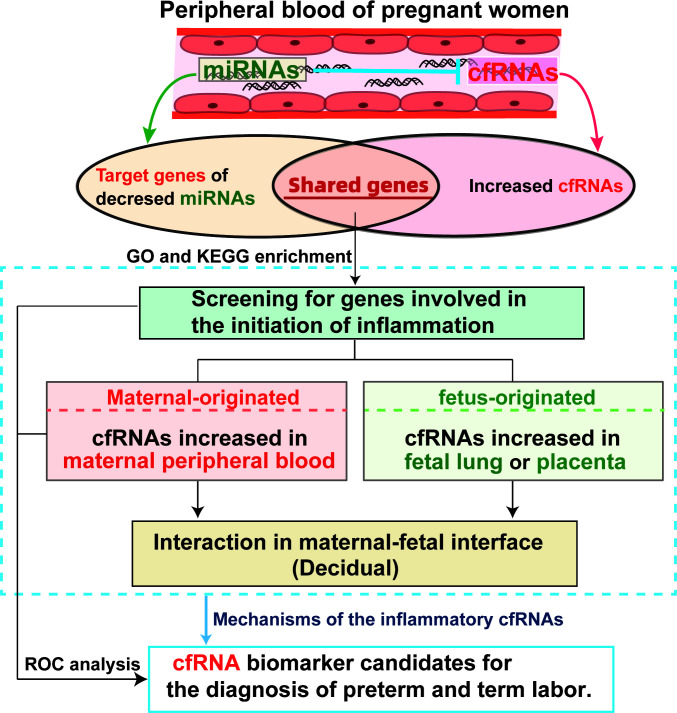
Diagram illustrating the analysis design and mechanisms involved in the study.

In the current study (**Figure 1**), we analyzed the inflammation related cfRNAs, which are released by maternal or fetal cells, and targets of exosome derived miRNA, which may be involved in the anti-inflammatory regulation in maternal blood at early gestation. As well as single-cell RNA transcripts of peripheral blood mononuclear cells from pregnant women, fetal lung, placenta, and decidua from pregnant women at different gestational ages were analyzed to locate the source and mechanism of screened cfRNAs. The goals of this study are to: (1) screen new biomarkers of cfRNA in maternal blood for the noninvasive prediction of preterm labor; (2) determine the origin of the potential cfRNA biomarkers; and (3) investigate the relationship between cfRNA biomarkers and maternal-fetal interface. The combination of cfRNA transcripts from pregnant peripheral blood and their developmental features in maternal or fetal side during gestation may help to discover novel biomarkers for noninvasive prediction and early diagnosis of preterm labor, as well as shed new light to our knowledge of inflammatory mechanisms underlying normal parturition and preterm labor.

## 2 Methods

### 2.1 Differential analysis of exosomal miRNA expression during gestation

The data for this study were collected from the Gene Expression Omnibus (GEO; https://www.ncbi.nlm.nih.gov/geo/) database and the European Bioinformatics Institute (https://www.ebi.ac.uk/) database.

To identify the dynamic changes of the exosomal miRNA in the maternal plasma across different gestational ages (first trimester, second trimester, third trimester, and delivery), full-term samples (**Table S1**) from dataset GSE115572 (10) were analyzed using the R package limma (v3.46.0). The miRNA that was significantly highly expressed in the samples of the first trimesters compared with later gestations (P <0.05) was defined as the differentially expressed miRNA. The validated target genes of the miRNA were collected with the R package multiMiR (v1.12.0). The differentially expressed miRNAs, and the intersections of the validated target genes and DEGs, were enriched with the R package clusterProfiler (v3.18.1).

### 2.2 Differential analysis of cfRNA expression during gestation

The normal samples (**Table S2**) from dataset (GSE192902) of cfRNA-sequencing from pregnant blood plasma across different gestation stages was used to analyze the differential expression of cfRNA during gestation (5). These samples have been classified into four groups, including “Early group” (gestation age < 12W, n=37), “Mid group” (gestation age between 13-20 weeks, n36), “Late group” (gestation age > 23 weeks, n=39), and “Postpartum group” (n=30). With these samples, we can analyze the cfRNA showed an increased trends in the relatively late gestation and decreased postpartum. The differential analysis was conducted based on the R package limma (v3.46.0), and the genes that were increased in samples from “Late group” with a P-value <0.05 were defined as the differentially expressed genes (DEGs). The visualizations of the KEGG (Kyoto Encyclopedia of Genes and Genomes) and GO (Gene Ontology) terms were conducted with ggplot2 (v3.3.6).

### 2.3 Data collection of single cell RNA-sequencing

The scRNA-seq data of maternal peripheral blood mononuclear cells were mined from databases GSM5761208, GSM5761210, and E-MTAB-6701 (22, 23). The scRNA-seq data of fetal lung were from database GSM2319567, GSM3027039, GSM3027047, GSM4504959, GSM5343154, GSM5343155, GSM4647254, and GSM4647255 (24–26). The scRNA-seq data of decidua were collected from databases E-MTAB-6701, GSM5646527, GSM5646530, GSM5646533, GSM5646536, GSM5646539, and GSM5646542 (23, 27). The scRNA-seq data of the placenta were collected from databases E-MTAB-6701, GSM5261695, GSM5261696, GSM5261697, and GSM5261698 (23, 28).

### 2.4 Data processing for scRNA-seq

The gene expression matrix was merged, and the batch effect was removed with the R package harmony v1.0 (lambda = 1, max.iter.harmony = 20). The merged expression matrix was prepared for clustering using Seurat (v4.1.0), following the common pipeline. The feature plots (based on R package Seurat) and dot-plot (based on the DotPlot function in R package ggplot2) were used to demonstrate the expression of marker genes.

### 2.5 Identification of differentially expressed genes in scRNA-seq data

The FindAllMarkers function in the R package Seurat was applied to identify differentially expressed genes for each cell cluster. The Wilcoxon Rank-Sum test was used to determine the significance of the difference (average Log2 FoldChange ≥ 0.25, adjusted P-value < 0.05). The feature plots and vioplots (based on R package Seurat) and the heatmap (based on R package pheatmap v1.0.12) were used to demonstrate the gene expression.

### 2.6 Calculation of M1-macrophage scores

AddModuleScore function in the R package Seurat was applied to the calculation of the gene expression modular scores per cell. The conventional classic activated macrophages (M1-subtype) modular included genes CCL5, CCR7, CXCL9, CXCL10, FCGR1A, FCGR1B, FCGR1C, HLADRA, IL12A, IL12B, IL23A, IL1A, IL1B, IL6, IL8, IRF1, IRF5, and TNF (29, 30).

### 2.7 The differentially expressed cfRNA between preterm and term labor

We analyzed the differentially expressed cfRNA transcripts (SRP130149) from peripheral blood of pregnant women who delivered preterm or term (2) using the FindAllMarkers function (DeSeq2) in Seurat after excluding the outliers. The information of the datasets was showed in **Table S3**. The visualization of differentially expressed cfRNA was performed using the boxplot function in R. ROC (Receiver Operating Characteristic) curve and area under the curve (AUC) values were computed with the R package ROCR (1.0-11) and pROC (1.18).

In addition, we also analyzed the target gene in the amniotic fluid cell-free transcriptome (GSE166956); the information of samples used in our analyses can be found in **Table S4** based on a t-test. The ROC curve and area under the curve (AUC) values were computed with the R package ROCR and pROC (1.18).

## 3 Results

### 3.1 Screening of increased cfRNAs associated with inflammation in late gestation pregnant blood

**Figure 2:**
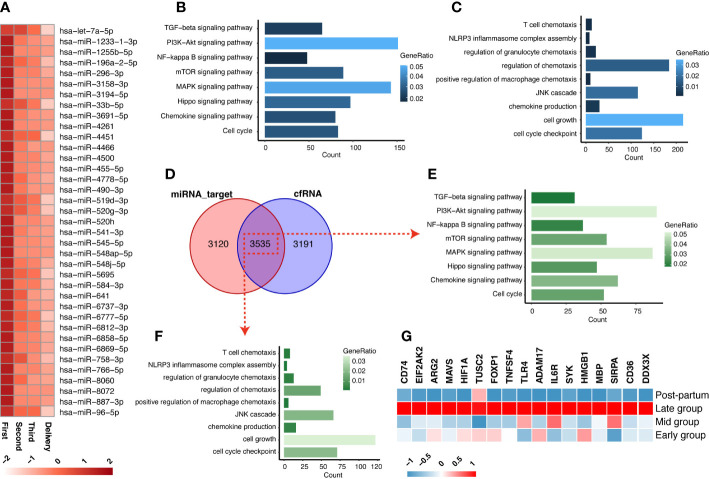
The exosomal miRNA and cfRNA in the maternal plasma across gestation. **(A)** The circulating exosomal miRNAs in the maternal plasma showed decreased trends during the gestation stage. **(B, C)** The KEGG **(B)** and GO **(C)** enrichments that were enriched from the target-genes of miRNA in decreased pattern. **(D)** The intersections between miRNA-targets and cfRNA (increased expressions in third trimester). **(E, F)** The KEGG **(E)** and GO **(F)** enrichments of the intersections of decreased miRNA-targets and increased cfRNAs. **(G)** The expressions of 17 screened inflammation-list genes across gestation.

The transition from anti-inflammation to pro-inflammation of pregnancy status is a key signal to the initiation of labor. miRNAs, as inhibited regulated factors to genes, play a vital role in the inhibition of inflammation in the early stage of pregnancy. Circulating exosomal miRNA can transfer to target cells and interfere with the expression of relative genes. Firstly, the dataset (GSE115572) of circulating exosomal miRNA in the maternal plasma across the 4 stages of gestation (first trimester, second trimester, third trimester, and delivery) was analyzed to identify the significant candidate miRNAs. As shown in **Figure 2A**, a total of 37 miRNAs were discovered to have decreased patterns from the first trimester stage to the late gestational stage. We further analyzed the target genes of these decreased miRNAs (**Table S5**), as well as how KEGG signaling pathways and GO analysis enriched the target genes. As shown in **Figures 2B, C**, the terms “chemokine production” and “NLRP3 inflammasome complex assembly” suggest that these decreased miRNAs may regulate the process of anti-inflammation during gestation.

Next, we investigated the increased cfRNAs, which may be released by inflammatory cells, in maternal peripheral blood during pregnancy by mining a cfRNA-sequencing dataset (GSE192902) from pregnant women at the corresponding gestational stages. A total of 3535 genes are shared between the cfRNAs that increased in the third trimester and the target genes of decreased miRNAs (**Figure 2D**; **Table S6**). We further screened the initiation of inflammation-related genes among the 3535 genes, which were enriched in the processes of chemokine production and inflammasome activation of macrophages with KEGG and GO enrichment analyses (**Figures 2D–F**). Finally, all the genes (CD74, EIF2AK2, ARG2, MAVS, HIF1A, TUSC2, FOXP1, TNFSF4, TLR4, ADAM17, IL6R, SYK, HMGB1, MBP, SIRPA, CD36, DDX3X) from the terms “chemokine production” and “NLRP3 inflammasome complex assembly”, 17 of the 3535 shared genes, are designated as inflammation-list and shown in **Figure 2G**. They may play a role in recruitment and activation of inflammatory cells associated with labor initiation and serve as potential cfRNA biomarkers for noninvasive early diagnosis of preterm labor.

### 3.2 TNFSF4 and ARG2 are abundantly expressed in pro-inflammatory macrophages from peripheral blood of late-gestational pregnant women

**Figure 3:**
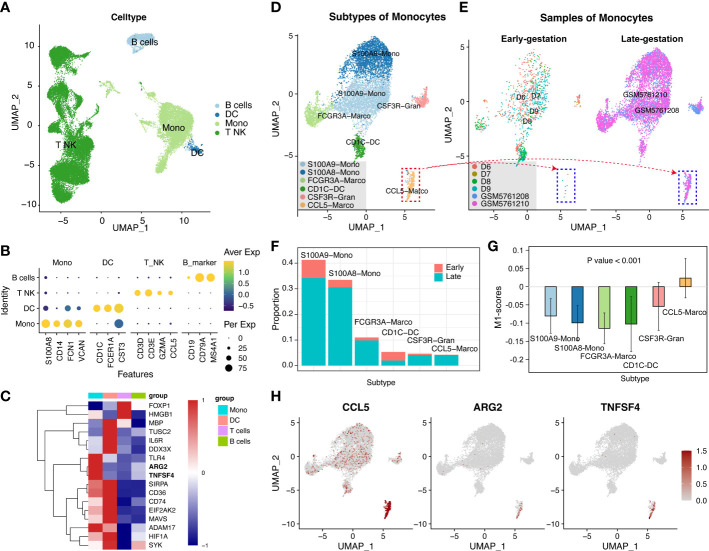
The peripheral blood mononuclear cells from pregnant women at late-gestation and early-gestation. **(A)** A UMAP-plot depicted profiled cells from pregnant women at late-gestation (28 to 32 weeks) and early-gestation (< 10 weeks). **(B)** Dot-plot depicted the expression of marker genes in each cluster. T and NK cells (CD3D, CD3E, GZMA, and CCL5), B cells (CD19, CD79A, and MS4A1), DC cells (CD1D, FCER1A, and CST3), and monocyte cells (S100A8, CD14, FCN1, and VCAN). **(C)** A heatmap of the genes in each cluster. **(D)** Myeloid lineage cell subclusters (Mono-cluster and DC-cluster). **(E, F)** The cell ratio of early- and late-gestation in each cluster. **(G)** The M1-scores for each cluster. **(H)** The expressions of CCL5, TNFSF4, and ARG2 in myeloid lineage cells.

To confirm the released cells involved and discover the mechanisms of these genes, the sources of the 17 candidate biomarkers in the peripheral blood of late-gestational pregnant women across the gestation should be identified. We firstly examined peripheral blood mononuclear cells from pregnant women before 10 weeks (early gestation; E-MTAB-6701) and between 28 and 32 weeks (late gestation; GSM5761208 and GSM5761210). Totally, 31659 single cells were obtained and classified with specific markers into T-NK cells (CD3D, CD3E, GZMA, and CCL5), B cells (CD19, CD79A, and MS4A1), DC cells (CD1D, FCER1A, and CST3), and monocyte cells (S100A8, CD14, FCN1, and VCAN) (**Figures 3A, B**). The expression pattern of the above 17 inflammation-list genes was shown in **Figure 3C**. Almost all the genes were expressed mainly in the myeloid cell cluster (monocyte cells and DC cells), except for the FOXP1 and HMGB1 which were predominantly expressed in the T-NK cell cluster. Given the role of monocytes and macrophages in the initial of labor, we mainly focus on myeloid cells that serve as a significant source of cfRNAs.

We further separated these myeloid cells into sub-clusters, i.e., CSF3R-Gran, S100A9-Mono, CD1C-DC, FCGR3A-Macro, S100A8-Mono, and CCL5-Macro (**Figures 3D**, **S1** and **Table S7**). Notably, the CCL5-Marco was only detected in late gestation (**Figures 3E, F**) and exhibited significantly high M1-scores **(**P<0.001**;** **Figure 3G**), indicating that it has pro-inflammatory features. Moreover, TNFSF4 and ARG2 showed specific expression in the CCL5-Macro cluster from late-gestational maternal peripheral blood mononuclear cells (**Figures 3H**, **S2**), indicating these two cfRNAs (TNFSF4 and ARG2) are maternally-originated and may function in an inflammatory process that initiates labor and delivery.

### 3.3 TNFSF4 receptor is upregulated in decidual T cells during late gestation

**Figure 4:**
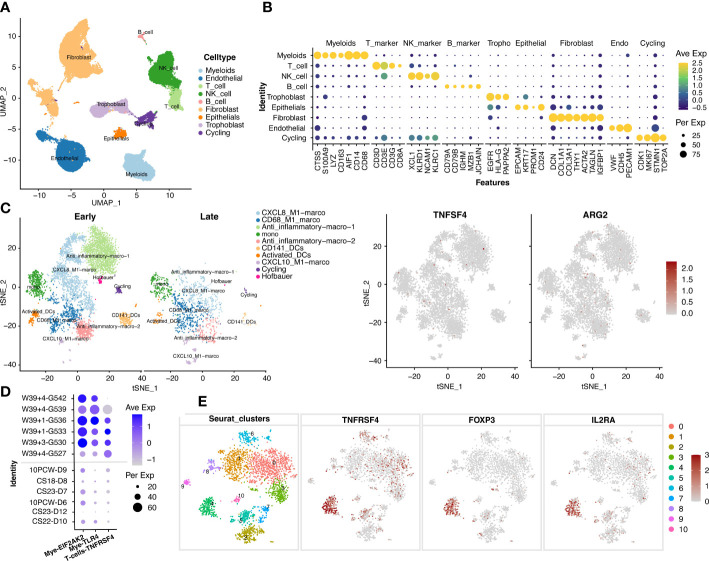
The increased expression of TNFSF4 receptor in T cells in decidual at late-gestation. **(A)** The cell types of 66617 single cells from decidual tissue in pregnant women. **(B)** A dotplot depicted the cell-type markers in deciduals. Endothelial cells (VWF, CDH5, PECAM1), fibroblast cells (DCN, COL1A1, COL3A1, THY1, ACTA2, TAGLN, IGFBP1), trophoblast (EGFR, HLA-G, PAPPA2), cycling cells (CDK1, MKI67, STMN1, TOP2A), epithelial cells (EPCAM, KRT17, PROM1, CD24), B cells (CD79A, CD79B, IGHM, MZB1, JCHAIN), myeloid cells (CTSS, S100A9, LYZ, CD163, AIF1, CD14, CD68), T cells (CD3D, CD3E, CD3G, CD8A), NK cells (XCL1, KLRD1, NCAM1, KLRC1). **(C)** The subtypes of myeloid cells and the expression of TNFSF4 and ARG2. **(D)** The expressions of TNFRSF4 (in T and NK cells), EIF2AK2 and TLR4 (in myloid cells) in the early-gestation (CS11-D10, CS23-D12, 10PCW-D6, CS23-D7, CS18-D8 and 10PCW-D9) and late-gestation (W39 + 4-G527, W39 + 3-G530, W39 + 1-G533, W39 + 1-G536, W39 + 4-G539, W39 + 4-G542). G527 indicates GSM5646527, G530 indicates GSM5646530, G533 indicates GSM5646533, G536 indicates GSM5646536, G539 indicates GSM5646539, and G542 indicates GSM5646542. **(E)** The sub-clusters of T cells and the expression of TNFRSF4 and Treg cell markers (IL2RA and FOXP3). CS, carnegie stage; W, week.

Given the important roles of the decidua serving as the maternal-fetal interface at the time of delivery, we further explore the mechanism that if the maternally-originated TNFSF4 and ARG2 interact with their target cells at decidua. The datasets of single cell RNA sequencing in the decidua before 10 weeks (E-MTAB-6701) and after 39 weeks (GSM5646527, GSM5646530, GSM5646533, GSM5646536, GSM5646539, and GSM5646542) were analyzed and obtained 66617 single cells. These single cells were classified with specific markers into endothelial cells (VWF, CDH5, PECAM1), fibroblast cells (DCN, COL1A1, COL3A1, THY1, ACTA2, TAGLN, IGFBP1), trophoblast cells (EGFR, HLA-G, PAPPA2), cycling cells (CDK1, MKI67, STMN1, TOP2A), epithelial cells (EPCAM, KRT17, PROM1, CD24), B cells (CD79A, CD79B, IGHM, MZB1, JCHAIN), myeloid cells (CTSS, S100A9, LYZ, CD163, AIF1, CD14, CD68), T cells (CD3D, CD3E, CD3G, CD8A), and NK cells (XCL1, KLRD1, NCAM1, KLRC1) (**Figures 4A, B**).We noted that TNFSF4 and ARG2 showed almost no expression in macrophages of decidua (**Figure 4C**). However, TNFRSF4, the receptor of TNFSF4, showed increased expression in the Treg cells (Foxp3+IL2RA+) from late-gestation compared to those from early-gestation (**Figures 4D, E**). This was consistent with the mouse decidua that the significantly expressed Tnfrsf4 was found in the T cells of mouse decidua (**Figure S3**), which suggested the inflammatory activities of T cells in decidual and eventually lead to delivery or even preterm labor.

### 3.4 Increased Eif2ak2 and Tlr4 cfRNAs are originated from fetal lung pro-inflammatory macrophages during late pregnancy

**Figure 5:**
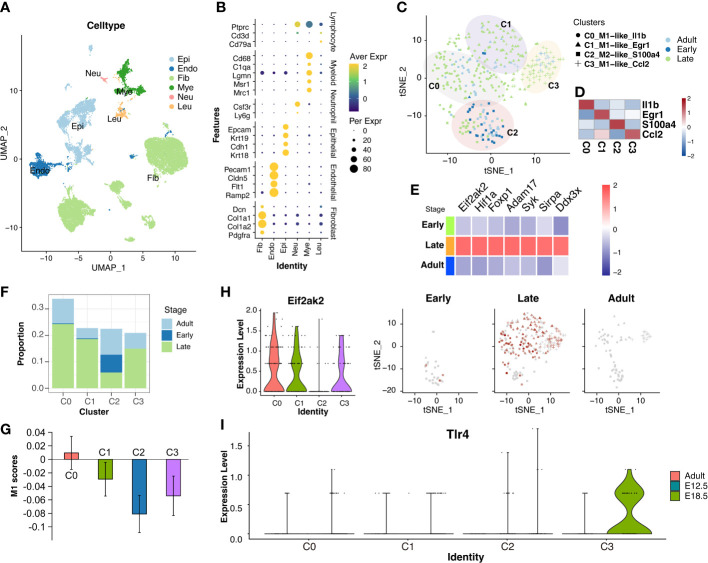
Increased Eif2ak2 and Tlr4 levels in pro-inflammation macrophages of the mouse fetal lung at late-gestation. **(A)** UMAP-plot showed profiled cells from fetal lung across gestation. **(B)** Dot-plot depicted the expression of marker gene in each cluster. The endothelial cells (Endo; Pecam1, Cldn5, Flt1, Ramp2), epithelial cells (Epi; Epcam, Krt19, Cdh1, Krt18), fibroblast (Fib; Dcn, Col1a1, Col1a2, Pdgfra), myeloid cells (Mye; Cd68, C1qa, Lgmn, Msr1, Mrc1), neutrophil cells (Neu; Csf3r, Ly6g), leukomonocyte cells (Leu; Ptprc, Cd3d, Cd79a). **(C, D)** Sub-clusters of macrophages **(C)** and expressions of the markers **(D)** in each cluster. **(E)** The expressions of inflammation-list gene were increased in late-gestation. **(F)** The ratio of cells from three gestations in the four sub-clusters in macrophages. **(G)** The M1-scores in the four sub-clusters in macrophages. the M1 scores of the C2 cluster were decreased compared to the rest cells with the P value of 0.02568. **(H, I)** The expressions of Eif2ak2 and Tlr4 in the four sub-clusters in macrophages.

Perhaps due to human ethical limitations, no more human embryonic datasets have been found within our capacity so far for us to analyze the origin of cfRNA. To further explore the tissue-origin of other inflammation-related cfRNAs that were increased during late gestation, the fetal lungs from pregnant mice at E9.5 (GSM2319567), E10.5 (GSM3027039), E12.5 (GSM3027047 and GSM4504959), and E18.5 (GSM5343154 and GSM5343155), as well as lungs from adult mice (GSM4647254 and GSM4647255), were included in this investigation. 23368 single cells were obtained and classified into endothelial cells (Endo; Pecam1, Cldn5, Flt1, Ramp2), epithelial cells (Epi; Epcam, Krt19, Cdh1, Krt18), fibroblast (Fib; Dcn, Col1a1, Col1a2, Pdgfra), myeloid cells (Mye; Cd68, C1qa, Lgmn, Msr1, Mrc1), neutrophil cells (Neu; Csf3r, Ly6g), and leukomonocyte cells (Leu; Ptprc, Cd3d, Cd79a) (**Figures 5A, B**).

Next, these macrophages from the fetal lung were classified into four sub-clusters (C0, C1, C2, and C3) (**Figures 5C, D**, **S4**), and the markers of C0, C1, and C3 showed M1-like characteristics (Il1b, Egr1, and Ccl2), and the marker of C2 showed M2-like characteristics (S100a4) (**Figure S5**). The TNFSF4 and ARG2 showed little expression in the macrophages from fetal lung, which further confirmed that these two cfRNAs are exclusively maternal-origin. However, another gene from the inflammation-list, i.e., Eif2ak2, was shown to increase in the macrophages during late gestation (**Figure 5E**). Among the four sub-clusters of macrophages, the C2 cluster included the highest ratio of cells from early-gestation, while the C0, C1, and C3 clusters included only a few cells from early-gestation (**Figure 5F**). Furthermore, the M1-scores in the C0, C1, and C3 were higher than those in the C2 (**Figure 5G**), which also suggested that these sub-clusters (C0, C1, and C3) have relatively more pro-inflammatory characteristics compared to the sub-cluster C2. Eif2ak2, whose expression was increased in macrophages at late gestation, was primarily expressed in C0, C1, and C3 macrophages (**Figure 5H**). Furthermore, another gene from the inflammation-list, Tlr4, was also increased in C3 macrophages during late gestation, although it did not show a significant increase in the bulk macrophages (**Figure 5I**). These results indicate that Eif2ak2 and Tlr4 were derived from pro-inflammatory macrophages in the fetal lungs of mice during late gestation.

### 3.5 EIF2AK2 and TLR4 were also increased in placental and decidual macrophages during late pregnancy

**Figure 6:**
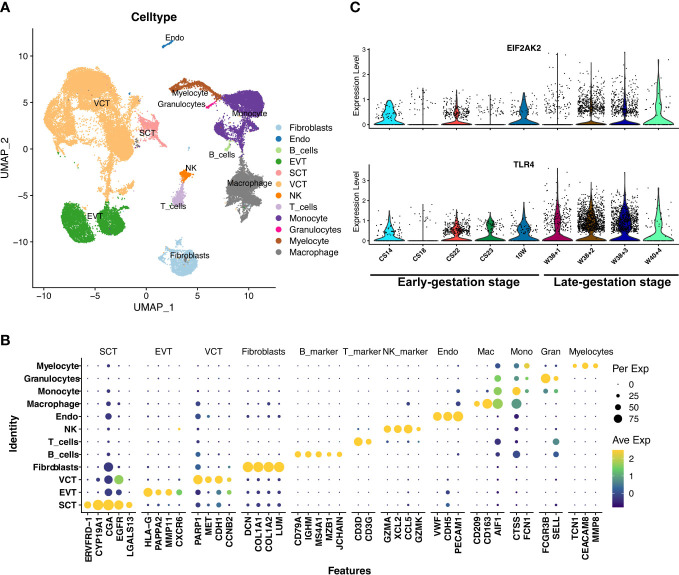
The expressions of EIF2AK2 and TLR4 in the placenta. **(A)** UMAP-plot showed profiled cells from the placenta across gestation. **(B)** The markers for celltyping. VCT indicates villous cytotrophoblast cells (PARP1, MET, CDH1, CCNB2), SCT indicates syncytiotrophoblast cells (ERVFRD-1, CYP19A1, CGA, EGFR, LGALS13), EVT indicates extravillous trophoblast cells (HLA-G, PAPPA2, MMP11, CXCR6), Fibroblasts indicates fibroblast cells (DCN, COL1A1, COL1A2, LUM), Endo indicates endothelial cells (VWF, CDH5, PECAM1), Macrophage indicates macrophage cells (CD209, CD163, AIF1), Monocyte indicates monocyte cells (CTSS, FCN1), Myelocyte indicates myelocyte cells (TCN1, CEACAM8, MMP8), and Granulocyte indicates granulocyte cells (FCGR3B, SELL), B_cells (CD79A, IGHM, MS4A1, MZB1, JCHAIN), T_cells (CD3D, CD3G), and NK_cells (GZMA, XCL2, CCL5, GZMK). **(C)** The EIF2AK2 and TLR4 were significantly (P<0.001) increased in macrophages of the placenta in late-gestation. CS14, CS18, CS22, CS23, 10W, W38 + 1, W38 + 2, W38 + 3, and W40 + 4 indicate the samples of different gestational ages. CS14, CS18, CS22, CS23 and 10W indicate the early-gestation stage before 10 weeks. W38 + 1, W38 + 2, W38 + 3 and W40 + 4 indicate the late-gestation stage after 38 weeks. CS, carnegie stage; W, week.

The placenta is another important fetal tissue and plays important roles in the inflammatory process at the maternal-fetal interface. So, we then analyzed the expression of inflammation-list genes in placenta before 10 weeks (E-MTAB-6701) and after 38 weeks (GSM5261695, GSM5261696, GSM5261697, and GSM5261698). The 40899 single cells were clustered into villous cytotrophoblast cells (PARP1, MET, CDH1, CCNB2), syncytiotrophoblast cells (ERVFRD-1, CYP19A1, CGA, EGFR, LGALS13), extravillous trophoblast cells (HLA-G, PAPPA2, MMP11, CXCR6), fibroblast cells (DCN, COL1A1, COL1A2, LUM), endothelial cells (VWF, CDH5, PECAM1), macrophage cells (CD209, CD163, AIF1), monocyte cells (CTSS, FCN1), myelocyte cells (TCN1, CEACAM8, MMP8), granulocyte cells (FCGR3B, SELL), B cells (CD79A, IGHM, MS4A1, MZB1, JCHAIN), T cells (CD3D, CD3G), and NK cells (GZMA, XCL2, CCL5, GZMK) (**Figures 6A, B**). We were surprised to see that the EIF2AK2 and TLR4 were also significantly increased in macrophages from placenta during late gestation (P<0.01) (**Figure 6C**) and decidua during late gestation (P<0.01) (**Figure 4D**). We also investigated the expression of Eif2ak2 and Tlr4 which were increased in the myeloid of mouse placenta along with pregnancy, which is consistent with the human placenta analysis (**Figure S6**). Thus, we speculated that the fetal-origins of EIF2AK2 and TLR4 maybe released by macrophages in fetal lungs and placenta, and the fetus-derived pro-inflammatory macrophages may migrate to the decidua and facilitate the term or preterm delivery.

### 3.6 The expression of TNFSF4 in the peripheral blood increased in preterm labor compared to term labor

**Figure 7:**
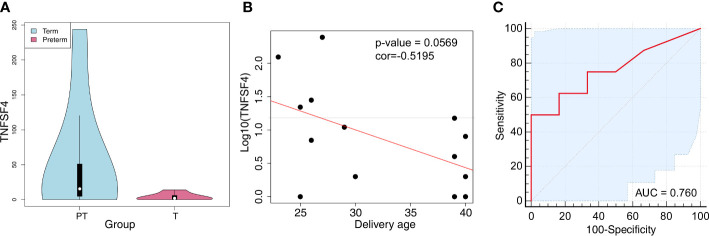
TNFSF4 expression in peripheral blood cfRNA of pregnant women with premature labor. **(A)** The expression of TNFSF4 in the peripheral blood cfRNA of pregnant women with premature or mature labor. P value<0.001, log2FC = 2.37. **(B)** The correlations of TNFSF4 expression and delivery age in the peripheral blood cfRNA of pregnant women. Correlation coefficient (r) = -0.5195, P-value = 0.0569. **(C)** The ROC (Receiver Operating Characteristic) curve and the area under the curve (AUC) values of TNFSF4 in peripheral blood cfRNA of pregnant women with premature labor. 95% CI: 0.4988-1.0 (DeLong).

As validation cohorts, we investigated the differentially expressed cfRNA transcripts between women delivered at preterm and full term (SRP130149) to confirm the potential of these cfRNAs as noninvasive biomarkers for preterm labor. The cfRNAs of TNFSF4 also showed significantly (P value < 0.001 and log2FC = 2.37) increased expression in women who delivered preterm (**Table S8**; **Figure 7A**). Among them, there was a negative relationship between the delivery age and the cfRNA of TNFSF4 (r = -0.5195; P value = 0.0569) (**Figure 7B**). The area under the curve (AUC) value of the ROC (receiver operating characteristic) curve is 0.76 (95% CI: 0.4988-1.0 (DeLong)) (**Figure 7C**). All these results indicated a relatively high efficiency of the cfRNA transcripts of TNFSF4 for the prediction of preterm delivery. In addition, we also investigated the cfRNA transcripts of TNFSF4 in amniotic fluid samples from women who delivered within 24 hours of amniocentesis compared to gestational age-matched samples from women who delivered after 24 hours of amniocentesis based on the transcriptome array. The analysis showed an increasing trend (P value = 0.0505) of TNFSF4 in amniotic fluid samples from women who delivered within 24 hours, and the ROC curve is 0.66 (95% CI: 0.3733-0.9378 (DeLong)) (**Figures S7A, B**). All these results further confirmed that the cfRNA transcript of TNFSF4 may serve as a promising noninvasive biomarker for preterm labor.

## 4 Discussion

Exosomes are involved in cell-to-cell signaling by carrying bioactive molecules, such as miRNAs and proteins (10, 12). Multiple reports have identified the usefulness of placental-derived exosomes miRNA in predicting the pregnancy outcomes (9–11). cfRNAs, non-invasive diagnostic biomarkers, have been found in pregnant women’s peripheral blood for the crucial purpose of predicting preterm birth (2). Most of the previous studies investigated the miRNAs or cfRNAs seperately. The combinational analysis of differentially expressed miRNAs and cfRNAs might provide more precise panels of non-invasive biomarkers for the prediction of preterm birth and help to further understand the biological processes that occurred during parturition, either term or preterm. In our integration analysis, we discovered 17 cfRNAs related to inflammation that were significantly increased at late gestation by overlapping the target genes of decreased expressed miRNAs and increased expressed cfRNAs from pregnant blood from early gestation to late gestation stages. Several of the 17 inflammation related cfRNAs have been reported to be associated with preterm birth in previous studies. For instance, HMGB1 (31) and TLR4 (3) have been confirmed and related to inflammation-induced preterm labor. But the origins of these cfRNAs, which increased during late gestation, have not been determined, which is significant to understanding the development of inflammation during pregnancy and parturition.

In present study, the majority of the 17 inflammation-related cfRNAs were expressed in the maternal peripheral blood myeloid cells, such as TLR4, ARG2, and TNFSF4. More interestingly, TNFSF4 and ARG2 had exclusive expression in the pro-inflammatory macrophage sub-cluster CCL5-Macro, which is specifically distributed in peripheral blood at late gestation, whereas no significantly differential expression was observed in macrophages of fetal lung between early and late gestation, or in the cells of the human (adult and fetal) lung (**Figure S8**). Thus, TNFSF4 has high potential as a candidate biomarker.

Furthermore, TNFRSF4, the receptor of TNFSF4, was also upregulated in the decidua at late gestation and plays an important role in T cell proliferation and functions. TNFRSF4 was reported to be mainly expressed by T cells at the site where inflammation and immune activity were activated (32, 33), which is consistent with our findings that TNFRSF4 expression is increased in Treg cells of the decidua. Tregs are critical drivers of maternal-fetal tolerance (34–37) and the dysfunction of Tregs has been implicated in preterm birth (38, 39). The inability of TNFRSF4-deficient T cells to reach sites of inflammation and reduce the effector functions (40, 41). Stimulation of Tregs by TNFSF4-TNFRSF4 interaction can reduce the capacity of Tregs (42). Additionally, the interaction between TNFSF4 and TNFRSF4 promoted the production of inflammatory molecules (40), such as CXCR4 (43) and RANTES/CCL5 (44). Although the expression of TNFRSF4 was not observed in preterm labor due to the lack of scRNA-seq data, the cfRNA of TNFSF4 was also found to increase in the maternal blood of preterm labor compared to term labor in the validation cohorts, suggesting that TNFSF4 is a promising non-invasive biomarker for preterm labor.

During the immunological tolerance of pregnancy, not only the maternal immune system but also the fetal immune system undergoes immunological adaptations. At late gestation, the inflammation-related cfRNAs Eif2ak2 and Tlr4 were found to be overexpressed in macrophages with increased inflammatory features from the fetal lung and placenta but not in maternal blood. As the firstly-emerged cells of the nascent immune system in embryonic development, macrophages differentiate into tissue-resident macrophages during the various stages of organ development (45). However, given the lung macrophages have not yet begun to specialize at the end of the embryonic stage (45), the macrophages detected in the fetal lung may reflect the features of circulating macrophages in the fetus. TLR4 is predominantly expressed in the macrophages of the placenta, which is consistent with our analysis (46, 47). Increased expression of TLR4 in the macrophages of the placenta villi was observed in the preterm patients (47).

Of interest, EIF2AK2 and TLR4 expression was also increased in macrophages of decidua at late gestation. Macrophages are critical components of the maternal–fetal interface; the pro-inflammatory phenotype of macrophages is required to initiate labor (48–50). EIF2AK2 activated the NLRP3 inflammasome and promoted the release of IL-1β and HMGB1 in macrophages, which were important for the activation of the inflammatory responses in macrophages (51, 52). As the receptor of HMGB1, the excessive activation of TLR4 can trigger intrauterine inflammation and promote the production of pro-inflammatory cytokines (IL-1, IL-6, IL-8, and chemokines) (3). Taken together, the increased expression of EIF2AK2 and TLR4 might subsequently or coordinately promote the M1-phenotype polarization of macrophages in the decidua at late gestation and enhance the inflammatory microenvironment at the maternal-fetal interface, which may stimulate uterine contraction and eventually lead to term labor or even preterm labor (23, 53, 54).

Due to the lack of human maternal and embryonic datasets, the limitation of this study is that we are unable to tell if the macrophages of decidua are tissue-resident or migrated from other fetal tissues, such as the fetal lung or placenta. In addition, the datasets of the amniotic fluid samples which was detected with the Affymetrix human transcriptome array platform which detects specific targets and has low detection sensitivity was not ideal to evaluate the cfRNA of TNFSF4. In the future, we will continue to follow this topic and confirm the more speculations surrounding this project, and the use of the fetal-specific markers to differentiate the origins of decidual macrophages is still warranted.

## 5 Conclusion

This study identified 17 cfRNAs related to initiation of inflammation that increased in the maternal blood during late gestation. Among them, EIF2AK2 and TLR4 were discovered to be overexpressed in pro-inflammation macrophages from the fetal lung, placenta, and decidua at late gestation, suggesting their potential fetal origin and functions of inflammatory activation at the maternal-fetal interface, which may trigger uterine contraction and contribute to the initiation of labor. TNFSF4 expression was elevated in pro-inflammatory macrophages of maternal blood but not in decidua at late gestation, whereas its receptor, TNFRSF4, was overexpressed in T cells of decidua during late gestation. These results suggested the maternal origin of TNFSF4, and the cell-cell communication between macrophages and T cells mediated by TNFSF4 and TNFRSF4 could initiate inflammatory status and contribute to the onset of labor as well. In addition, the cfRNA of TNFSF4 was significantly increased in maternal blood from preterm labor compared to that from term labor. Our findings not only uncovered a novel promising biomarker for noninvasive detection of preterm labor but also helped to understand the common characteristics shared by term and preterm labor, i.e., inflammatory process at the maternal-fetal interface, and shed new light on the early diagnosis and treatment of preterm delivery.

## Data availability statement

The original contributions presented in the study are included in the article/**Supplementary Material**, further inquiries can be directed to the corresponding author/s.

## Author contributions

Conceptualization: ZW, LG. Methodology: ZW, QO. Investigation: ZW, QO. Supervision: LG. Writing—original draft: ZW, QO. Writing—review and editing: ZW, QO, LG. All authors contributed to the article and approved the submitted version.

## Supplementary material

Supplementary Figure 1 Feature plots demonstrating the expression of selected markers on the umap-plot in the peripheral blood myeloid cells from pregnant women.

Supplementary Figure 2 Feature plots indicating the expression of the 15 screened cfRNAs in the peripheral blood myeloid cells from pregnant women.

Supplementary Figure 3 Feature plots indicating the expression of Tnfsf4 in the mouse decidua. **(A)** UMAP-plot indicated the cell types of mouse decidua. **(B, C)** The expression of the Tnfsf4 **(B)** and Tnfrsf4 **(C)** in the mouse decidua with feature plot. **(D)** UMAP-plot indicated the T cell clusters of mouse decidua. **(E)** The expression of the Tnfsf4 in T cells of the mouse decidua with feature plot.

Supplementary Figure 4 Feature plots demonstrating the expression of selected markers on the tSNE-plot in the macrophages from fetal lung.

Supplementary Figure 5 Feature plots demonstrating the expression of selected markers for four subclusters on the tSNE-plot in the macrophages from the fetal lung.

Supplementary Figure 6 Feature plots indicating the expression of selected markers in the immune cells of the mouse placenta at multiple stages of pregnancy.

Supplementary Figure 7 TNFSF4 expression in amniotic fluid cfRNA of pregnant women with premature labor. **(A**) The increased trends of TNFSF4 expression in amniotic fluid cfRNA of women who delivered within 24 hours compared to those after 24 hours. P-value = 0.0505. **(B)** The ROC curve and the area under the curve (AUC) values of TNFSF4 in amniotic fluid cfRNA of women who delivered within or after 24 hours. 95% CI: 0.3733-0.9378 (DeLong).

Supplementary Figure 8 Feature plots indicating the expression of TNFSF4 in the adult and fetal lung.

Supplementary Table 1 The information of exosomal miRNA sample datasets across the gestations.

Supplementary Table 2 The information of cfRNA sample datasets from pregnant blood plasma across different gestation stages.

Supplementary Table 3 The related information and the levels of cfRNA TNFSF4 for the preterm and full-term pregnant women.

Supplementary Table 4 The information and the levels of cfRNA TNFSF4 of samples in the amniotic fluid cell-free transcriptome analysis.

Supplementary Table 5 The targeted genes of the 37 decreased miRNAs across the gestations.

Supplementary Table 6 The genes are overlapped between the cfRNAs that increased in the third trimester and the target genes of decreased miRNAs.

Supplementary Table 7 The differential expressed genes of subtypes of myeloid cells in peripheral blood from pregnant women.

Supplementary Table 8 The differentially expressed cfRNAs of the peripheral blood of women between preterm and term labor.
