# Supplementary figures and images for: The increased cfRNA of TNFSF4 in peripheral blood at late gestation and preterm labor: its implication as a noninvasive biomarker for premature delivery
Source: Front Immunol. 2023 May 18;14:1154025. doi: 10.3389/fimmu.2023.1154025 (PMC10232964; doi:10.3389/fimmu.2023.1154025)

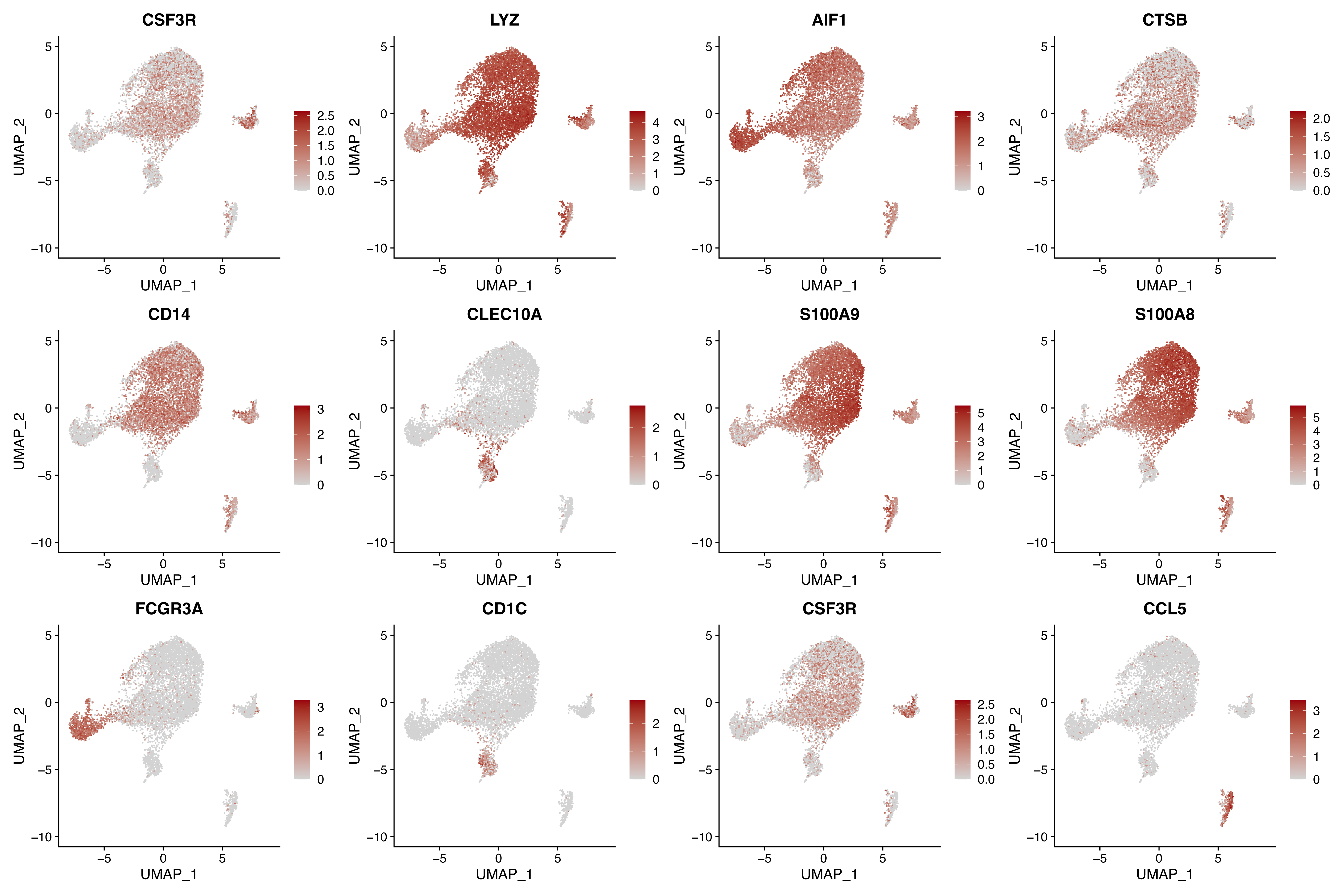

Supplement: Supplementary Figure 1 — Feature plots demonstrating the expression of selected markers on the umap-plot in the peripheral blood myeloid cells from pregnant women. [file Image_1.jpeg]

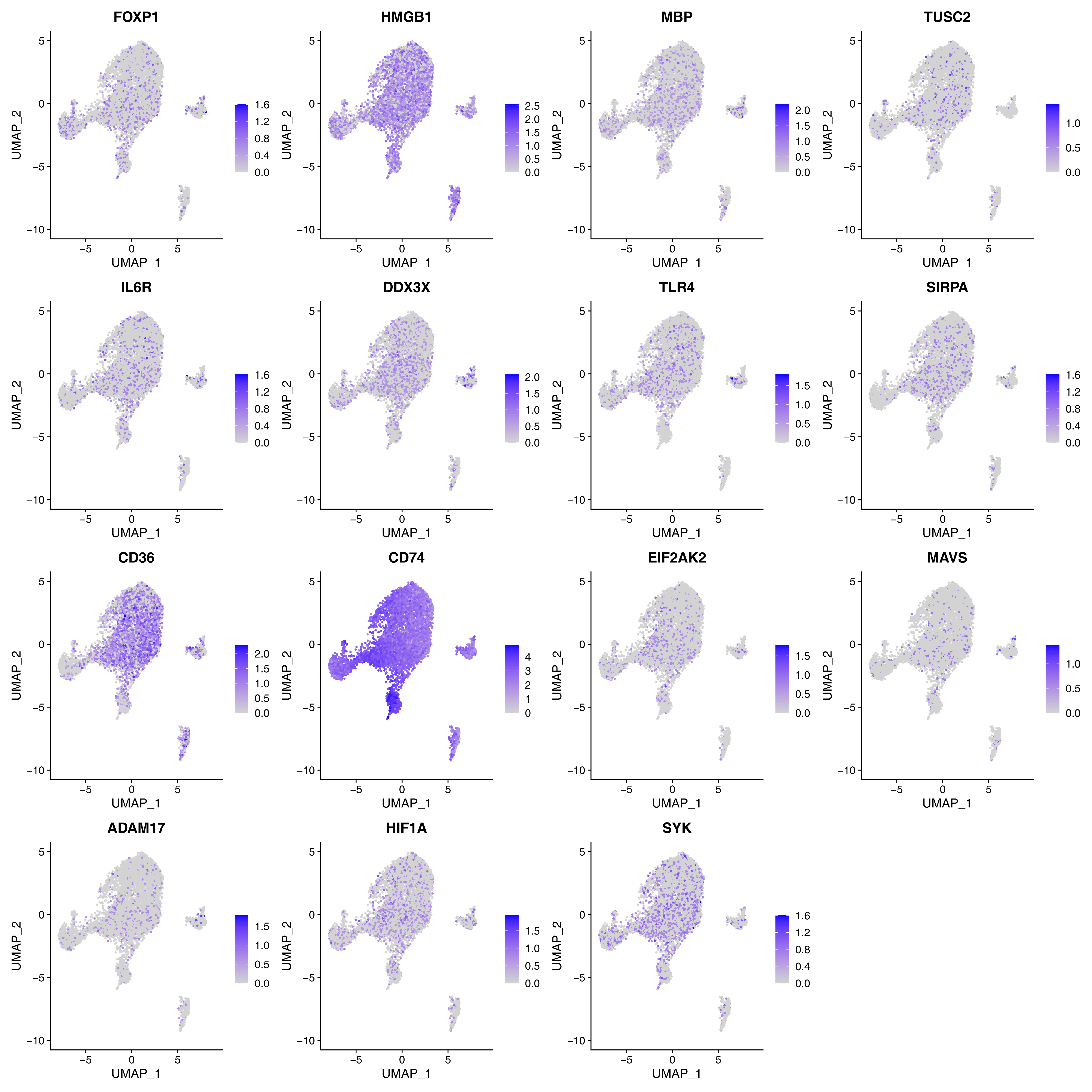

Supplement: Supplementary Figure 2 — Feature plots indicating the expression of the 15 screened cfRNAs in the peripheral blood myeloid cells from pregnant women. [file Image_2.jpeg]

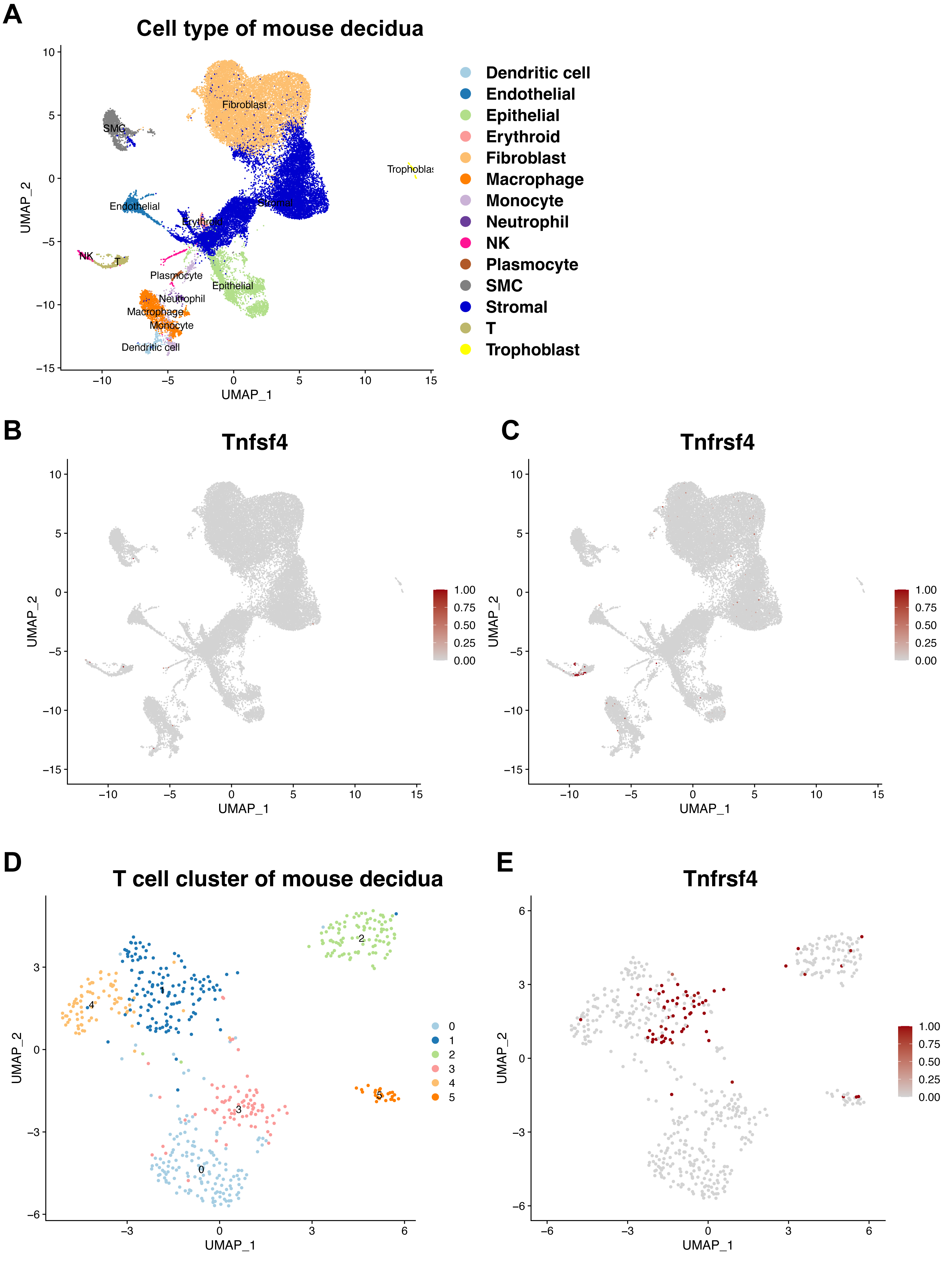

Supplement: Supplementary Figure 3 — Feature plots indicating the expression of Tnfsf4 in the mouse decidua. (A) UMAP-plot indicated the cell types of mouse decidua. (B, C) The expression of the Tnfsf4 (B) and Tnfrsf4 (C) in the mouse decidua with feature plot. (D) UMAP-plot indicated the T cell clusters of mouse decidua. (E) The expression of the Tnfsf4 in T cells of the mouse decidua with feature plot. [file Image_3.jpeg]

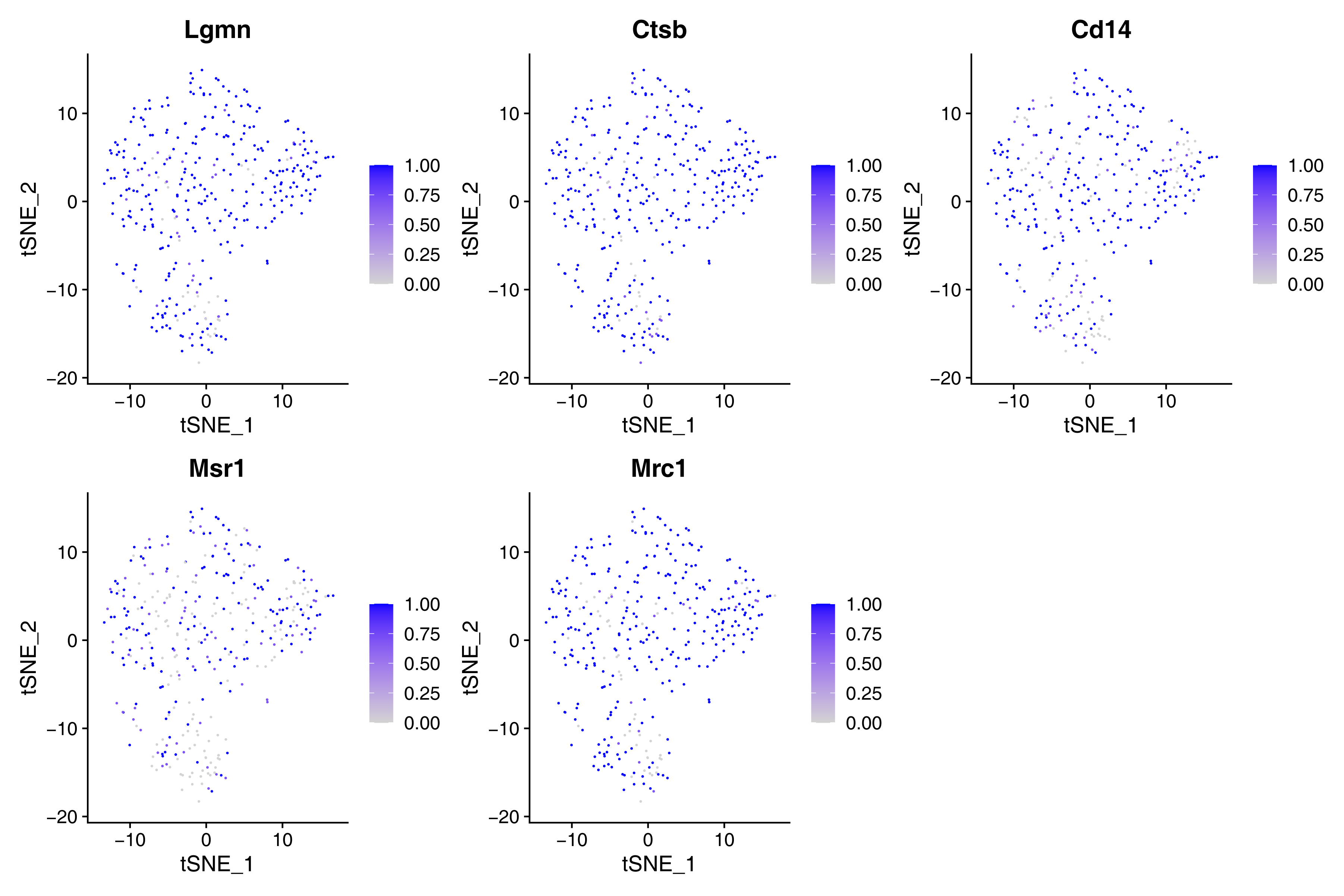

Supplement: Supplementary Figure 4 — Feature plots demonstrating the expression of selected markers on the tSNE-plot in the macrophages from fetal lung. [file Image_4.jpeg]

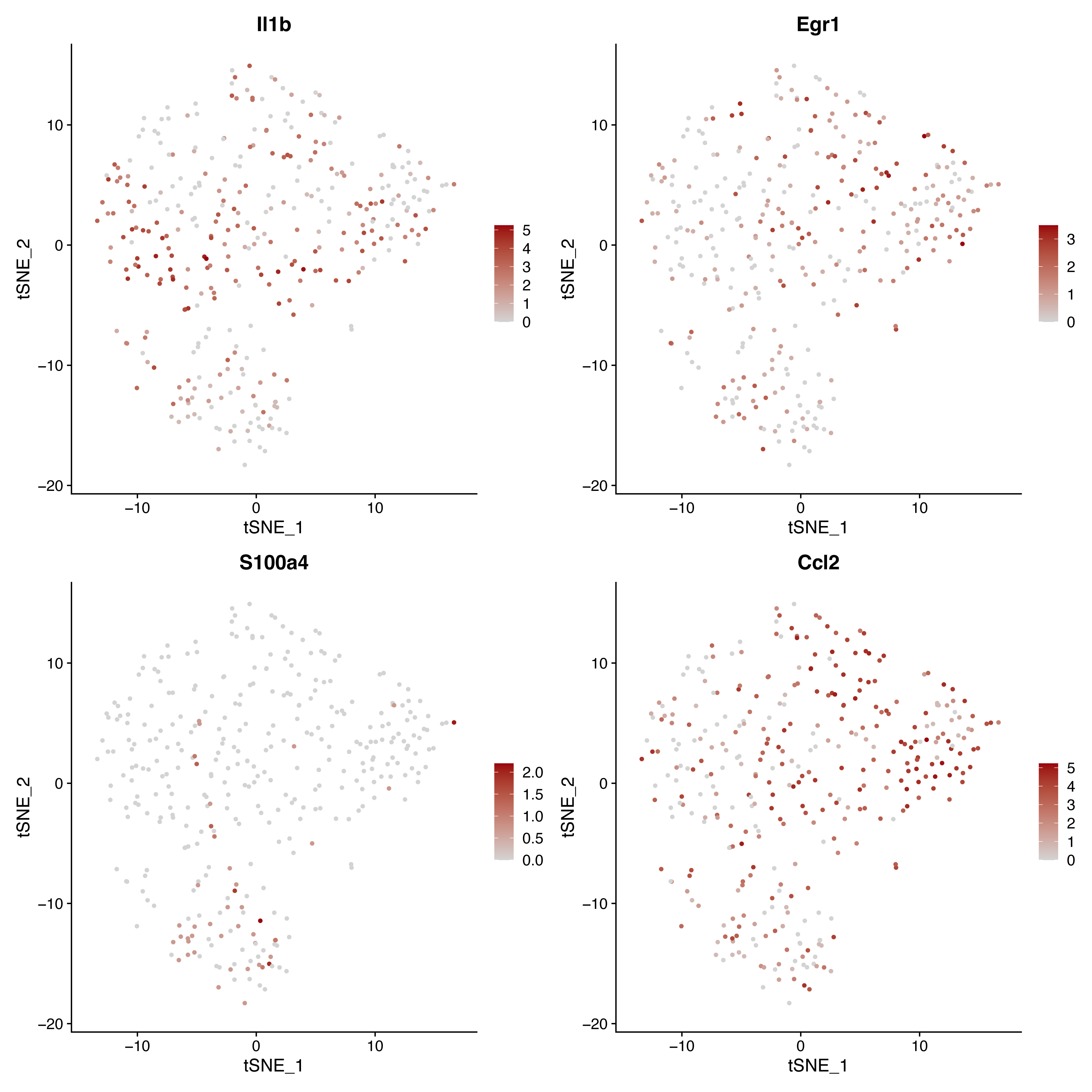

Supplement: Supplementary Figure 5 — Feature plots demonstrating the expression of selected markers for four subclusters on the tSNE-plot in the macrophages from the fetal lung. [file Image_5.jpeg]

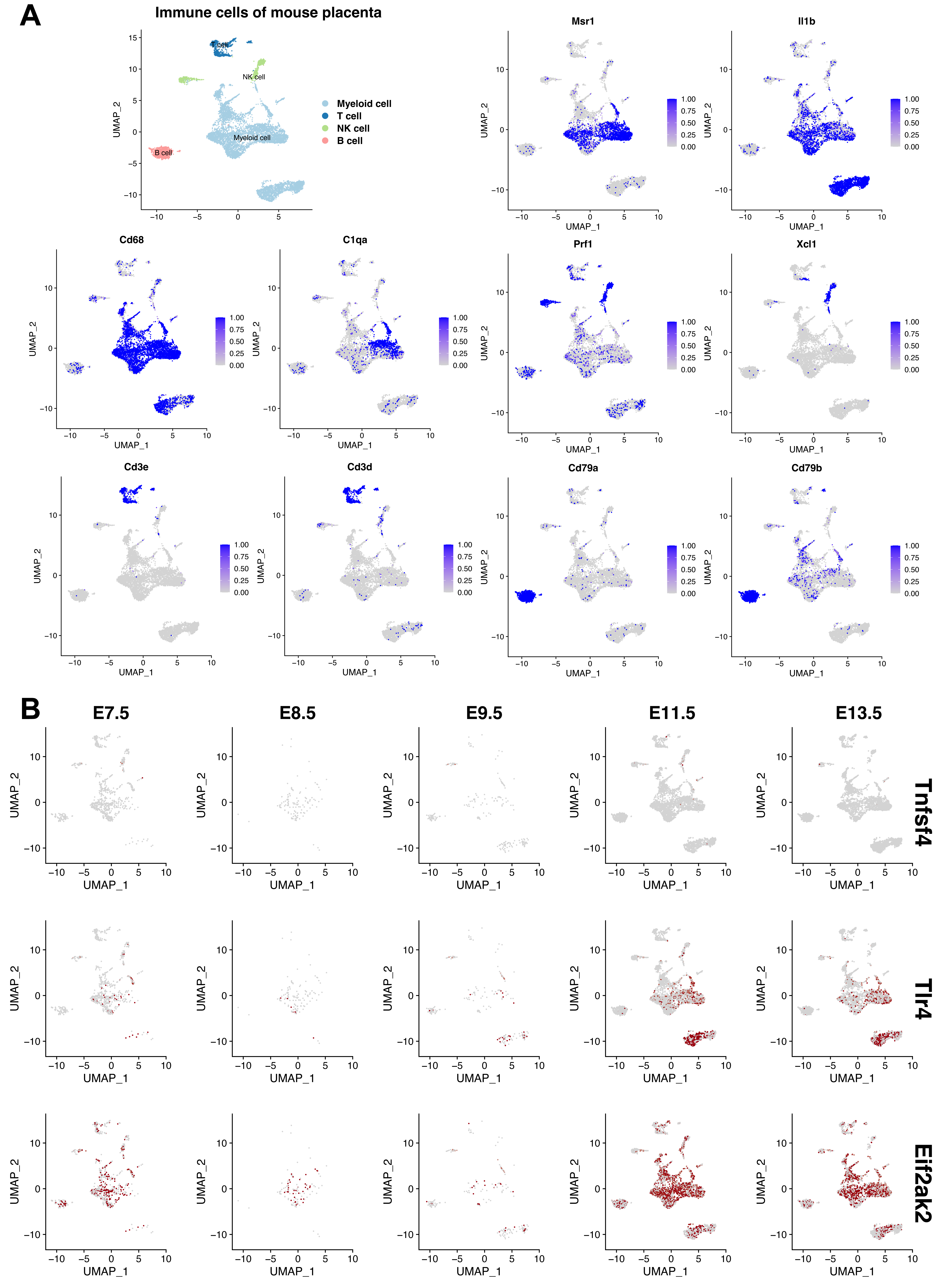

Supplement: Supplementary Figure 6 — Feature plots indicating the expression of selected markers in the immune cells of the mouse placenta at multiple stages of pregnancy. [file Image_6.jpeg]

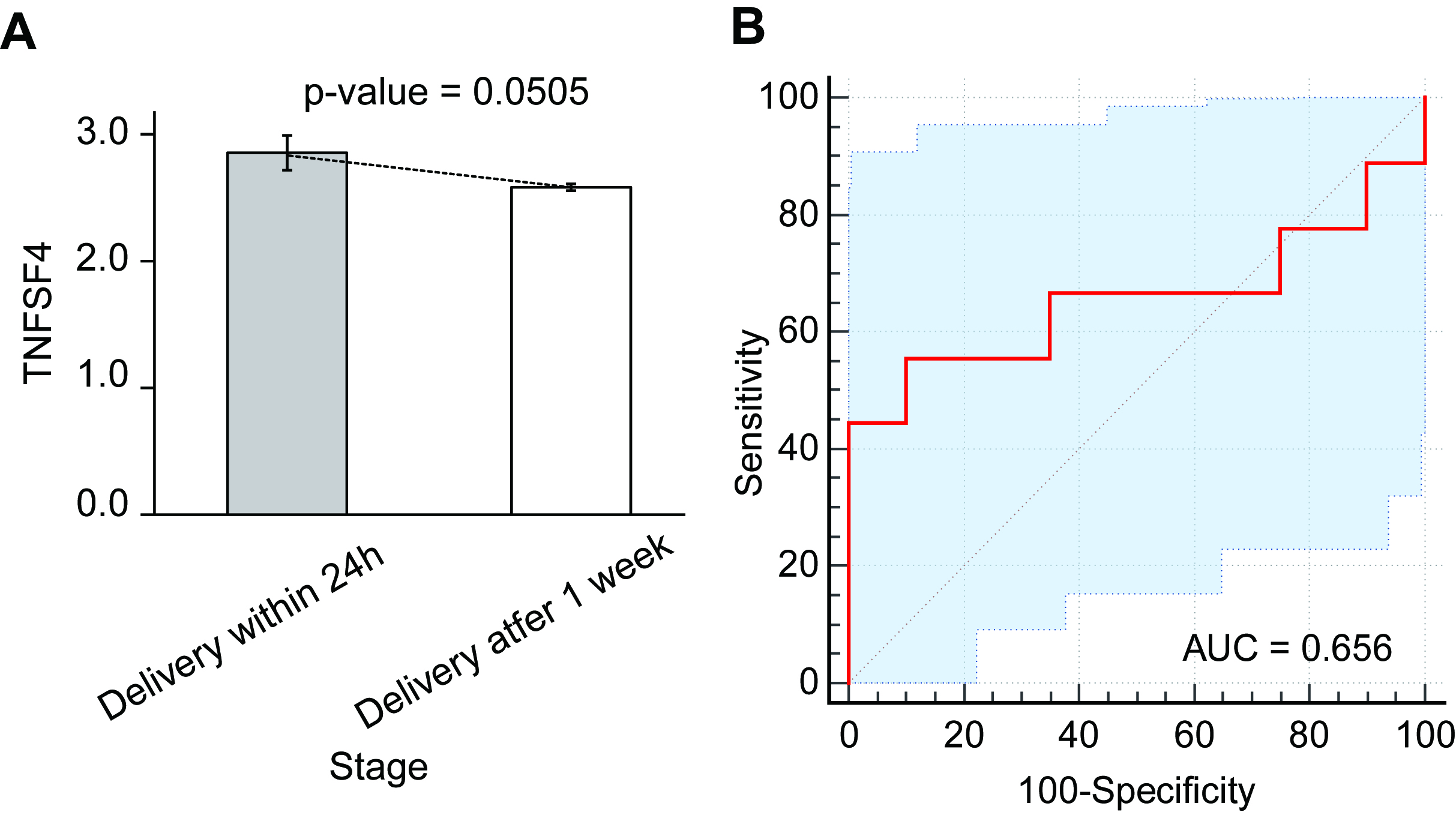

Supplement: Supplementary Figure 7 — TNFSF4 expression in amniotic fluid cfRNA of pregnant women with premature labor. (A) The increased trends of TNFSF4 expression in amniotic fluid cfRNA of women who delivered within 24 hours compared to those after 24 hours. P-value = 0.0505. (B) The ROC curve and the area under the curve (AUC) values of TNFSF4 in amniotic fluid cfRNA of women who delivered within or after 24 hours. 95% CI: 0.3733-0.9378 (DeLong). [file Image_7.jpeg]

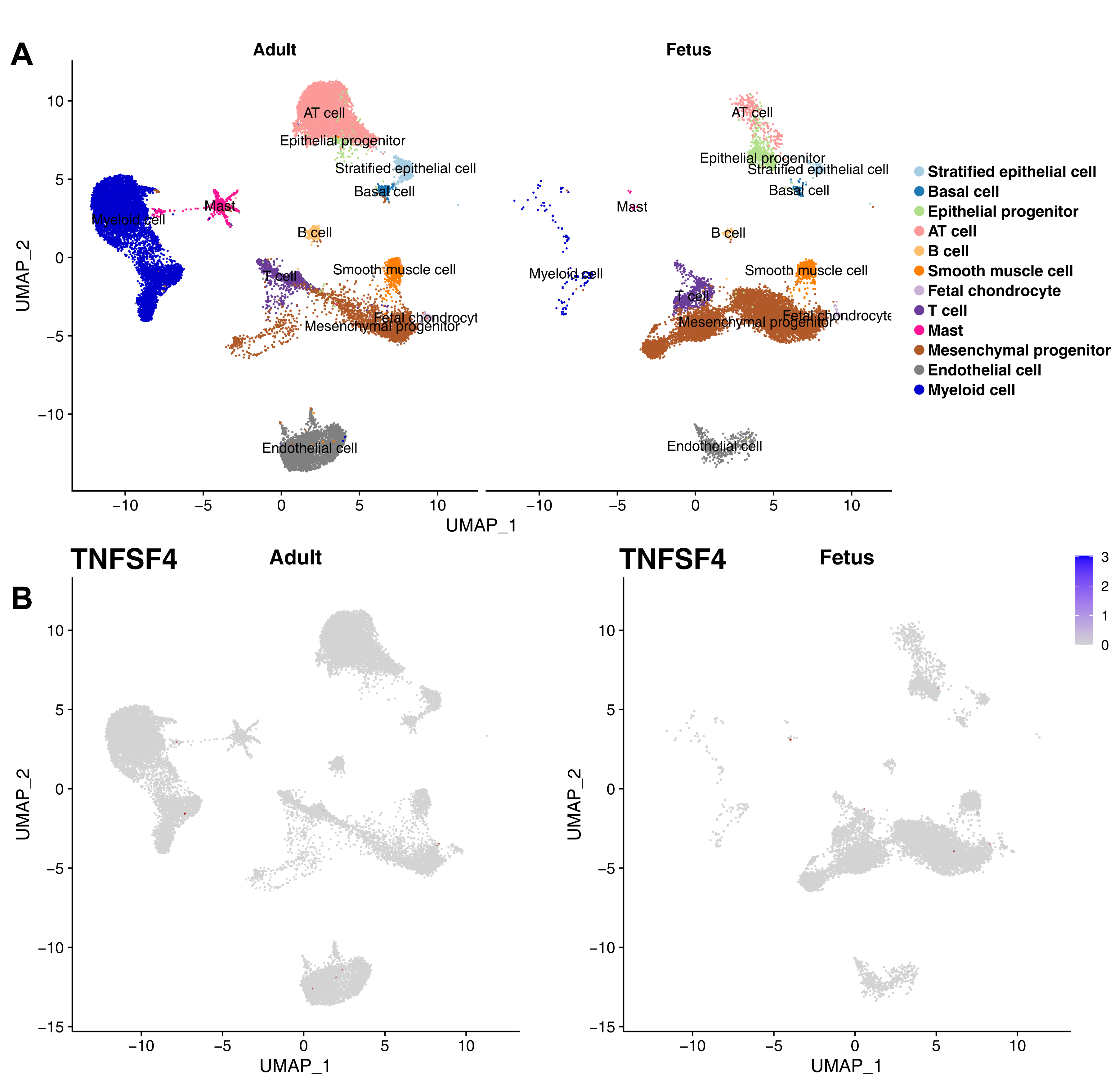

Supplement: Supplementary Figure 8 — Feature plots indicating the expression of TNFSF4 in the adult and fetal lung. [file Image_8.jpeg]
